# An Intriguing Case of Delirium: Recognizing Steroid-Responsive Encephalopathy Associated With Autoimmune Thyroiditis

**DOI:** 10.7759/cureus.70447

**Published:** 2024-09-29

**Authors:** Devipriya Surapaneni, Noel Sam, Sharath Chandra Dasi, Bhubaneshwar N

**Affiliations:** 1 Internal Medicine, Saveetha Medical College and Hospitals, Saveetha Institute of Medical and Technical Sciences, Chennai, IND; 2 Internal Medicine, Saveetha Medical College and Hospitals, Saveetha Institute of Medical and Technical Sciences, chennai, IND

**Keywords:** acute delirium, hashimoto’s encephalopathy, hashimotos thyroiditis, pulse dose steroids, steroid-sparing agent

## Abstract

Steroid-responsive encephalopathy, also known as Hashimoto’s encephalopathy, is an uncommon autoimmune neuroendocrine disorder linked to thyroiditis. It presents a variable range of clinical symptoms, making it challenging to diagnose. SREAT (steroid-responsive encephalopathy associated with autoimmune thyroiditis) is considered a diagnosis of exclusion. Diagnosing SREAT poses a challenge as its symptoms can overlap with those of acute conditions such as encephalitis and meningitis, as well as various autoimmune disorders and issues related to substance toxicity and abuse, all of which must be ruled out prior to establishing a definitive diagnosis and starting treatment. However, high anti-TPO antibodies (anti-thyroid peroxidase) in serum and rarely in the CSF favor the diagnosis of the condition. Although rare, Hashimoto's encephalopathy should be considered when managing cases of unexplained encephalopathy. Treatment of choice in these sets of patients includes high-dose corticosteroid therapy. We present a case of Hashimoto's encephalopathy in a middle-aged woman who arrived at our emergency department with altered mental status and facio-brachial seizures. After excluding various potential causes of acute encephalopathy, a diagnosis of SREAT was made. Testing of serum and cerebrospinal fluid (CSF) indicated the presence of anti-thyroid peroxidase (anti-TPO) antibodies, which is uncommon in CSF. Following intravenous steroid therapy, the patient experienced substantial improvement, reinforcing the diagnosis.

## Introduction

Steroid-responsive encephalopathy associated with autoimmune thyroiditis (SREAT), known as Hashimoto's encephalopathy, is a rare neurological condition marked by neurological and psychiatric symptoms along with elevated levels of anti-thyroid antibodies [1]. Diagnosing SREAT poses a challenge due to its nonspecific clinical presentation, which can overlap with symptoms of other neurological disorders. Standard laboratory investigations and neuroimaging studies are not definitive for this condition. The estimated incidence of SREAT is generally considered to be around 2 per 100,000 people per year. This estimate is based on clinical observations and case reports. The condition is more commonly reported in women, with a higher prevalence among middle-aged individuals. However, it can affect people of any age or gender.

The first documented case of Hashimoto's encephalopathy (HE) was reported in 1966 by Lord Brain and his colleagues. Since then, many cases have been noted, demonstrating a broad spectrum of clinical presentations [1].

The onset of SREAT can be sudden and severe. It is characterized by episodes of cerebral hypoperfusion, seizures, episodes of psychosis, etc. Alternatively, SREAT may insidiously present with depressive symptoms, cognitive dysfunction, movement disorders such as myoclonus and tremors, and fluctuating levels of consciousness [2]. Delirium, characterized by acute confusion, disorientation, and altered consciousness, is a relatively common symptom in patients with SREAT, as was the primary presentation in our patient. Delirium has been reported in approximately 20% to 40% of SREAT patients. SREAT can resemble other neuropsychiatric disorders, making a high degree of suspicion necessary for accurate diagnosis. It is important to include SREAT in the differential diagnosis, as timely treatment can lead to the reversal of neurological symptoms [3].

Seizures are also common neurological manifestations in patients with Hashimoto's encephalopathy (SREAT), most seen in its acute form. The reported incidence of seizures in these patients varies across studies, generally ranging from 50% to 70%. Our patient presented with an alleged history of suspected facio brachial seizures. Seizures may be managed temporarily with antiepileptics such as phenytoin. However, in certain instances, seizures may not respond to standard anti-seizure medications and could show greater improvement with steroid therapy [4].

The association between autoimmune thyroiditis and encephalopathy remains an intriguing and not fully understood aspect of autoimmune neurology. The pathogenesis of SREAT is still uncertain. The autoimmune nature of this disease is generally supported by several factors: a higher prevalence in females, the unpredictable and variable course of the illness, and its association with other autoimmune disorders.

While the detection of anti-TPO antibodies in the cerebrospinal fluid (CSF) is rare in SREAT, it is a significant finding that strongly reinforces the diagnosis, as evidenced by our patient. Several potential mechanisms in the pathophysiology of SREAT have been suggested, including humoral factors, immune complex formation, vasculitis, intrathecal synthesis of anti-thyroid antibodies, and global cerebral ischemia, as described by Pattanagere Manjunatha et al. [5]. 

The primary treatment for SREAT is high-dose corticosteroid therapy, often administered intravenously initially for a rapid response, followed by oral tapering of the dose. This treatment is based on the observation that many patients experience significant improvement in their symptoms within 48 hours of steroid initiation. Other treatment approaches may include immune modulators, intravenous immunoglobulin (IVIG), and plasma exchange. While these treatments show promise, there are no established guidelines for SREAT treatment. A rapid and significant improvement in neurological symptoms following the initiation of corticosteroid therapy is considered a hallmark of SREAT. Our patient showed a dramatic response after initiation of pulse steroid therapy, thus supporting the diagnosis.

This case report highlights the clinical presentation, diagnostic challenges, and therapeutic management of a patient with Steroid-Responsive Encephalopathy Associated with Autoimmune Thyroiditis (SREAT) who uniquely presented with positive anti-thyroid peroxidase (anti-TPO) antibodies in the cerebrospinal fluid (CSF). The detection of anti-TPO antibodies in CSF is exceedingly rare, as most cases of SREAT primarily report antibody positivity in serum, making this case particularly valuable in expanding the diagnostic considerations for SREAT. The rarity of CSF anti-TPO positivity underscores the diagnostic challenges in such cases, where the presence of antibodies in CSF may indicate a more direct or localized autoimmune response within the central nervous system, a phenomenon not well-documented in the literature. This report adds to the limited data available on the relationship between neuroimaging findings, thyroid status, autoantibody profiles, and brain pathology in SREAT, particularly emphasizing the diagnostic utility of CSF analysis.

## Case presentation

A 52-year-old female patient arrived at our emergency department with a sudden onset of altered mental status that had developed over the past 24 hours. The pattern of her encephalopathy was intermittent, with periods of complete normalcy and the ability to carry out daily activities between episodes. A detailed history taken from the patient's relatives revealed that she had experienced intermittent episodes of acute confusional states. During these episodes, she became aggressive and disoriented, with each episode lasting a few minutes and resolved spontaneously.

The patient’s history includes an episode of brief jerky movements affecting the face and right upper limb, lasting for one minute and witnessed by her husband. This episode resolved spontaneously, but it was unclear whether similar episodes had occurred previously. Additionally, there was a noted history of bed-wetting during an acute confusional state and trouble sleeping for the past two days. Based on this history, a presumptive diagnosis of delirium, epilepsy, and possible faciobrachial dystonic seizures was considered. The patient reported no history of fever, headache, loss of consciousness, trauma, falls, visual disturbances, sensory or motor deficits, or respiratory or gastrointestinal symptoms. She had no prior psychiatric diagnoses or history of substance abuse, and her family history was not significant. Personal history indicated that she is a known diabetic, hypertensive, and hypothyroid patient, though she has not been compliant with her medications. Specific medication details were not provided.

On examination in the hospital, the patient was conscious, oriented, obeying commands, and had stable vital signs. Systemic examination revealed no neurological deficits and other systems were unremarkable. Fundoscopy revealed no abnormalities. The Mini-Mental State Examination (MMSE) score was 28, and the Glasgow Coma Scale (GCS) score was 15/15. However, during episodes of delirium, the patient exhibited aggression and was disoriented to time, place, and person; therefore, the MMSE could not be assessed during those episodes. Throughout her hospital stay, the patient continued to have trouble sleeping and experienced similar episodes of agitation. Initially, her acute neuropsychiatric symptoms were not attributed to organic pathology due to the lack of evidence. After consulting with the psychiatry department, the patient was placed on daily follow-up and counseling, with antipsychotic medications used to control the episodes of delirium.

**Figure 1:**
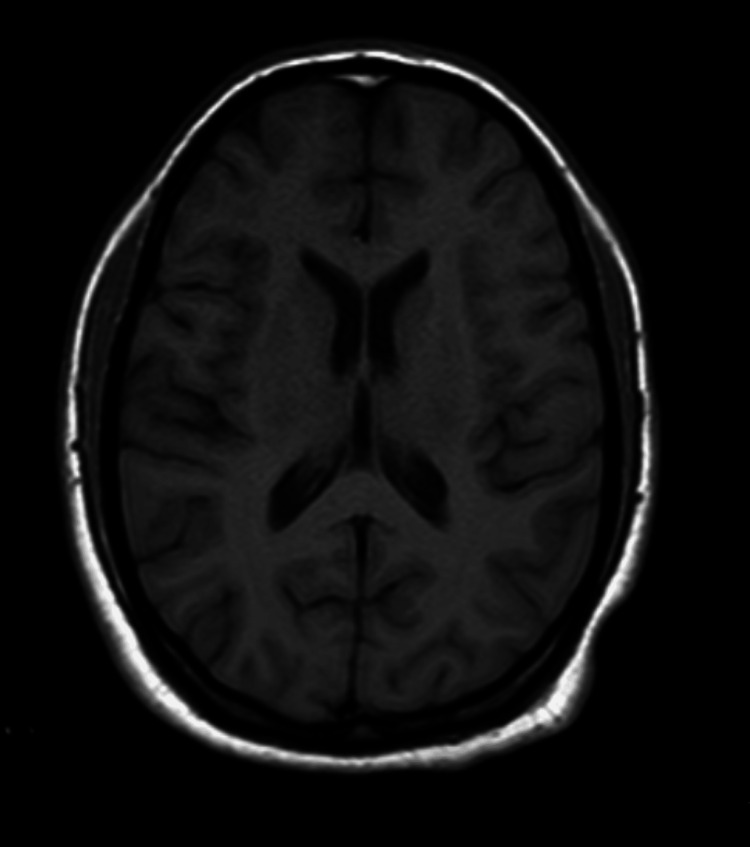
Normal contrast enhanced MRI brain No significant intracranial was abnormality noted.

Routine laboratory investigations revealed hyponatremia, with a serum sodium level of 121 mg/dL (Table 1). Thyroid function tests showed elevated TSH (thyroid stimulating hormone) and low free T3 (triiodothyronine) and T4 (thyroxine) (Table 2). The patient was initially managed with thyroid supplements, fluids, and supportive measures. A contrast-enhanced MRI of the brain showed no significant abnormalities (Figure 1). However, her symptoms were initially attributed to hyponatremia, the lack of improvement after treating the metabolic abnormalities led to the reconsideration of a potential underlying psychiatric disorder.

**Table 1:** Laboratory investigations on the day of admission. The complete blood count was within normal limits as shown above.

INVESTIGATION	LAB VALUE	REFERENCE RANGE
Hemoglobin (Hb) (G/Dl)	12.8	(Male 13-17, Female 12-15)
Total Rbc Count (Million/Cu.Mm)	4.7	(Male 4.5-5.5, Female 3.8-4.8)
Packed Cell Volume (Pcv) (%)	44.3	(Male 40-50, Female 36-46)
Mean Corpuscular Volume (Mcv) (Fl)	81.2	83-101
Mean Corpuscular Hemoglobin (Mch) (Pg)	29.5	27-32
Mean Corpuscular Hemoglobin Concentration (Mchc) (G/Dl)	34.4	31.5-34.5
Red Cell Distribution Width (%)	12.5	11.6-14
Platelet count (lakhs / mm3)	2.65	1.5-4.5
Total Leucocyte Count (Tlc) (Cells/Cumm)	9060	4000-10000
Neutrophils (%)	58.6	40-80
Lymphocytes (%)	36.1	20-40
Monocytes (%)	5	2-10
Eosinophils (%)	0.9	1-6
Basophils (%)	<1-2	0-4

**Table 2:** Serum electrolytes on day of admission and thyroid profile of the patient. The above investigations revealed hyponatremia and hypothyroidism.

INVESTIGATIONS	LAB VALUES	REFERENCE RANGE
Serum Sodium (Meq/L)	121	137-145
Serum Potassium (Meq/L)	3.5	3.5-5
(F-T3) Free Triiodothyronine (pg/ml)	1.77	2.77 - 5.27
(F-T4) Free Thyroxine (ng/dl)	0.87	0.78 - 2.19
(TSH)Thyroid Stimulating (mIu/ml)	19.7	0.46-4.68
Anti-TPO Antibody (IU/ml)	792.64	<=5.61

Suspecting septic encephalopathy, blood cultures were sent and returned negative. A lumbar puncture revealed lymphocytic predominance, high protein, and normal glucose in the CSF (Table 3). Given the suspicion of viral encephalitis, antiviral medications (acyclovir) and steroids were initiated, but there was no significant improvement. The CSF autoimmune encephalitis panel (anti-NMDA receptor, anti-LGI1, anti-CASPR2, anti-GABAB receptor, anti-AMPA receptor) was also negative (Table 3). Testing for serum and CSF anti-TPO antibodies showed highly elevated anti-TPO antibodies in both samples (Table 3). The detection of these antibodies, especially in cerebrospinal fluid (CSF), is highly sensitive and specific for the diagnosis of Hashimoto’s encephalopathy.

**Table 3:** CSF analysis report

CSF	LAB VALUE	REFERENCE RANGE
Total Count (cells/cu.mm)	147 cells	0-5
Neutrophils (%)	0	
Lymphocytes (%)	100	
Glucose (mg/dl, GOD-POD method)	159	50-80
Protein (mg/dl, precipitation method)	72.15	15-60
ADA (IU/L, Enzymatic method)	6.80	<10
CBNAAT	NEGATIVE	
Cytology	NEGTAIVE	
Auto Immune Encephalitis Panel	NEGATIVE	
CSF ANTI-TPO (U/ml)	16.5	>10

The patient was diagnosed with SREAT after excluding other possible causes for her symptoms. The patient was treated with intravenous pulse steroids, receiving 1 gram of intravenous methylprednisolone for three consecutive days. Within 48 hours, a dramatic improvement in the patient's sensorium and overall condition was observed. She was discharged with a tapering dose of oral steroids and continued regular follow-up. No relapses were seen, and her improvement was significant.

## Discussion

Hashimoto's encephalopathy is a neuropsychiatric disorder defined by symptoms like cognitive impairment, episodes resembling stroke, seizures, and mood disorders, which may include psychosis [6]. Associated with autoimmune thyroiditis, it is particularly distinguished by its significant responsiveness to steroid therapy, which is why it is also known as steroid-responsive encephalopathy associated with autoimmune thyroiditis (SREAT). The exact prevalence of SREAT is not well established due to underdiagnosis and misdiagnosis. SREAT is linked to autoimmune thyroiditis, including Hashimoto's thyroiditis, and is frequently associated with positive serum anti-thyroid antibodies, including anti-thyroid peroxidase (anti-TPO) and anti-thyroglobulin antibodies. There are no specific geographical or ethnic predispositions documented, indicating that SREAT can manifest across diverse populations worldwide.

Hashimoto’s encephalopathy exhibits a broad clinical spectrum. Patients often present with cognitive decline [7], memory impairment, confusion, and altered consciousness. Psychiatric symptoms such as mood disturbances, psychosis, impulsiveness, intense paranoia [8,9], and hallucinations are also reported. The first case of HE was reported in 1966 by Lord Brain and his colleagues, where the patient displayed symptoms including tremors, hallucinations, and confusion. Since then, many cases have been reported in the literature, the incidence being 2.1 people per 100,000 in the general population [1]. However, this case is exceptionally rare, as only a few instances of positive anti-TPO antibodies in cerebrospinal fluid (CSF) have been documented in the literature, with an estimated incidence of less than 10% in SREAT patients, emphasizing the need for further investigation into the connection between thyroid autoimmunity and central nervous system involvement.

According to Chong et al., the majority of SREAT cases display a fluctuating course with a myriad of symptoms ranging from cognitive decline to neuropsychiatric manifestations. Focal neurological deficits have also been noted. The incidence of seizures [10,11] in patients with steroid-responsive encephalopathy associated with autoimmune thyroiditis (SREAT) can range from 30% to 50%. Specifically, faciobrachial dystonic seizures (FBDS), which were a notable feature in our patient, are characterized by facial and arm twitching. These seizures have been documented in a subset of SREAT patients, with an incidence of around 10%, making them quite rare. FBDS often indicates an underlying autoimmune process rather than other probable etiologies, which posed an initial diagnostic dilemma in our case [12].

The clinical features of SREAT are not directly influenced by an individual's thyroid status. Although most patients with SREAT are euthyroid, there are documented cases in individuals with both overt hyperthyroidism and overt hypothyroidism, as observed in our patient who was hypothyroid. Elevated anti-TPO antibodies in serum are frequently observed in patients with SREAT but seldom in cerebrospinal fluid, which is the unique feature highlighted in this case report. Their presence in CSF supports the diagnosis, especially when neuropsychiatric symptoms are present and other causes have been excluded. While anti-TPO antibodies are not exclusive to SREAT and can be found in other autoimmune thyroid diseases, their detection in the context of neurological symptoms is highly suggestive of SREAT. According to Mocellin et al. [13], thyroid function typically appears normal in individuals with SREAT, both clinically and biochemically. Elevated anti-TPO antibodies are recognized as defining characteristics of SREAT. In fact, high concentrations of anti-TPO-Ab are found in nearly all documented cases, highlighting them as a critical marker for diagnosing the condition. Anti-thyroglobulin antibodies are detected in 45-60% of cases.

The presence of anti-TPO antibodies in cerebrospinal fluid is a rare finding with an incidence of less than 10% and a strong indicator of SREAT, confirming the diagnosis. Although the exact contribution of thyroid autoantibodies to CNS damage remains unclear, they primarily function as diagnostic markers. A study by Ferracci et al. demonstrated that antithyroid antibodies and circulating immune complexes were found in the CSF of six individuals diagnosed with Hashimoto's encephalopathy (HE), while these markers were not present in the CSF of 21 control subjects. The production of autoantibodies and CIC occurred within the central nervous system, and their levels did not correlate with the patients' medical condition or treatment regimen. Detecting these markers in the CSF of individuals experiencing acute or subacute encephalopathy shows promise as a diagnostic tool for HE [14].

Analysis of cerebrospinal fluid (CSF) usually shows elevated protein levels, a slight increase in lymphocytes (known as lymphocytic pleocytosis), and normal glucose concentrations. The detection of oligoclonal bands in the CSF may suggest an autoimmune or inflammatory condition affecting the central nervous system. However, it is important to emphasize that CSF protein levels and lymphocyte count within their normal range do not exclude the possibility of a Hashimoto's encephalopathy diagnosis [5]. The presence of anti-thyroid peroxidase (anti-TPO) antibodies in the CSF, while not exclusive to SREAT, is significant and contributes to the diagnosis, especially when correlated with serum antibody levels and the clinical response to steroid treatment.

In a systematic review led by Holanda et al that explored the clinical manifestations, laboratory results, cerebrospinal fluid (CSF) findings, and patient outcomes in individuals with Hashimoto's encephalopathy, CSF analysis revealed a predominant elevation in protein levels, detected in 71.1% of the patients. Among the 20 patients assessed for anti-TPO antibodies in the CSF, 15 individuals (75%) tested positive for these antibodies [2].

The criteria for diagnosing steroid-responsive encephalopathy associated with autoimmune thyroiditis (SREAT), as outlined by Graus and his team, highlight the presence of encephalopathy that has either an acute or subacute onset. Additionally, these criteria emphasize the importance of assessing thyroid function and the response to steroid treatment as integral components of the diagnosis. The patient should have additional symptoms such as hallucinations, seizures, stroke-like episodes, etc. When it comes to neuroimaging, especially MRI, the findings should either appear normal or exhibit non-specific abnormalities. It is vital to ensure that there are no detectable antineuronal antibodies in either the serum or the cerebrospinal fluid (CSF), such as anti-NMDA receptor antibodies, anti-AMPA receptor antibodies, anti-GABA-B receptor antibodies, anti-LGI1 antibodies, anti-CASPR2 antibodies, anti-CV2/CRMP5 antibodies, and anti-GAD65 antibodies. These are commonly associated with various forms of autoimmune encephalitis and paraneoplastic neurological syndromes [15]. Additionally, it is important to systematically eliminate other possible explanations for the patient's presenting symptoms [16].

While there are no standardized guidelines for treating SREAT, common therapeutic approaches include intravenous or oral corticosteroids, immune modulators such as azathioprine, methotrexate, and cyclophosphamide, as well as IVIG (intravenous immunoglobulin). In this case, the patient experienced a significant improvement following intravenous steroid therapy, a common clinical observation in SREAT. Other treatment options include intravenous immunoglobulin and plasma exchange. While the use of steroid responsiveness as a diagnostic criterion for SREAT is not universally accepted, a positive response to steroids strongly suggests the involvement of autoimmunity in the development of the disease.

## Conclusions

The above case report emphasizes the difficulties in diagnosing and managing SREAT, a neurologic condition characterized by a diverse range of symptoms, including cognitive impairment, seizures, and delirium, often associated with autoimmune thyroiditis, particularly Hashimoto's thyroiditis. The presence of elevated anti-TPO antibodies in CSF, while not exclusive to SREAT, is a significant indicator and supports the diagnosis. While a definitive diagnosis is often challenging due to the non-specific nature of its clinical presentation, prompt treatment with corticosteroids often leads to a rapid improvement in symptoms, underscoring the autoimmune basis of the condition. The case report also focuses on the importance of considering delirium as a common presentation of SREAT, given its high prevalence in affected individuals. 
